# Altered inflammatory response in FMRP-deficient microglia

**DOI:** 10.1016/j.isci.2021.103293

**Published:** 2021-10-15

**Authors:** Jennifer M. Parrott, Thomas Oster, Hye Young Lee

**Affiliations:** 1 The Department of Cellular and Integrative Physiology, The University of Texas Health Science Center at San Antonio, San Antonio, TX, USA

**Keywords:** Molecular biology, Neuroscience, Immunology

## Abstract

Fragile X syndrome (FXS) is an inherited intellectual disability with a high risk for comorbid autism spectrum disorders. Since FXS is a genetic disease, patients are more susceptible to environmental factors aggravating symptomatology. However, this confounding interaction between FXS environmental and genetic risk factors is under-investigated. Here, *Fmr1* knock-out (KO) mice and the immune stimulus lipopolysaccharide (LPS) were used to explore this interaction between FXS development and inflammation in microglia, the brain’s primary immune cell. Our results demonstrate that *Fmr1* KO and wild-type (WT) microglia are not different in inflammatory outcomes without LPS. However, *Fmr1* KO microglia produces an elevated pro-inflammatory and phagocytic response following LPS treatment when compared to WT microglia. Our experiments also revealed baseline differences in mitochondrial function and morphology between WT and *Fmr1* KO microglia, which LPS treatment exaggerated. Our data suggest an altered inflammatory mechanism in *Fmr1* KO microglia implicating a gene and environment interaction.

## Highlights

• *Fmr1* KO microglia display elevated LPS-induced pro-inflammatory gene expressions
• *Fmr1* KO microglia display elevated LPS-induced pro-inflammatory cytokine releases
• *Fmr1* KO microglia demonstrate increased LPS-induced phagocytic responses
• *Fmr1* KO microglial mitochondria have altered properties and LPS-stimulated responses

## Graphical abstract

**Figure:**
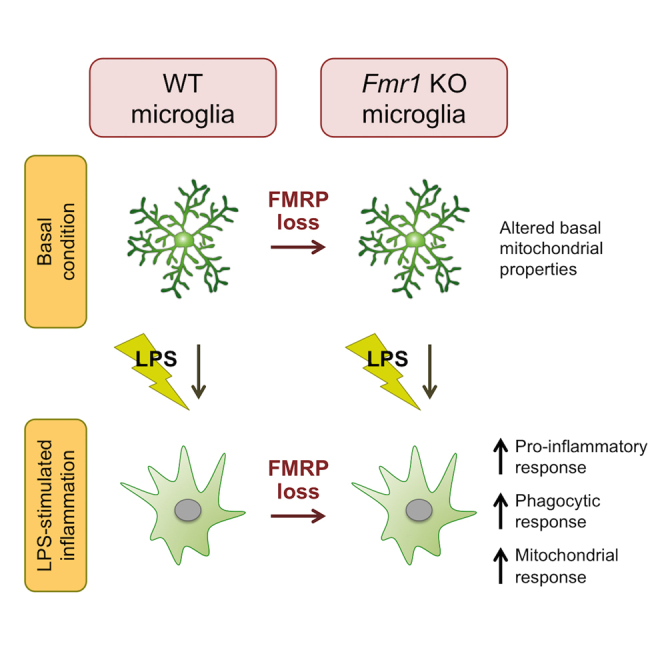

## Introduction

Autism spectrum disorder (ASD) is a group of neurodevelopmental disorders that has an estimated prevalence of approximately 62 in 10,000 worldwide (Elsabbagh et al., 2012). Much ASD research has explored underlying genetic causes resulting in the description of numerous genes that contribute to the risk of developing ASD (Betancur, 2011; Persico and Napolioni, 2013). However, these genetic mutations are heterogeneous and account for only 10–20% of ASD cases in patients (Betancur, 2011). Environmental risk factors have also been implicated in the development of ASD, both in idiopathic ASD and in potential interactions with susceptibility genes already present (Chen et al., 2015; Modabbernia et al., 2017). Environmental risk factors that have been identified include prenatal and postnatal immune stimulation, heavy metal toxicity, and pregnancy-related and birth-related complications (Modabbernia et al., 2017; Morris et al., 2017; Rossignol and Frye, 2012). However, in the context of ASD development, this confounding interaction between environmental and genetic risk factors has been under-investigated. Studies have demonstrated that ASD patients have a dysregulated immune system, including both peripheral immune system activation as well as abnormal microglial activation and elevated cytokine levels (Koyama and Ikegaya, 2015; Nakagawa and Chiba, 2016; Napolioni et al., 2013; Rossignol and Frye, 2014; Theoharides et al., 2013). As microglia are the resident immune cells in the brain, they would be involved in both an altered basal neuroimmune environment and an exaggerated response to incoming inflammatory signals (González-Scarano and Baltuch, 1999). Neuroinflammation is typically characterized by cytokine production, chemokine secretion, phagocytosis, and reactive oxygen species released primarily by microglia. Inflammatory responses are also associated with changes in mitochondrial dynamics, including mitochondrial membrane potential, mitochondrial size (through fusion or fission), and reactive oxygen species production (Di Filippo et al., 2010). During prolonged neuroinflammatory conditions, microglia can become overstimulated resulting in neuronal death (Boje and Arora, 1992; Bal-Price and Brown, 2001). Preclinical models have demonstrated that an elevated or prolonged neuroinflammatory response can result in the development of autistic behaviors (Fernández de Cossío et al., 2017). In ASD patients, infection or injury-induced inflammation could act as an environmental risk factor, interacting with the underlying genetic mutations that contribute to disease development, thereby worsening disease progression.

Fragile X syndrome (FXS) is a genetic intellectual disability (ID) in which patients often display autistic features (21–50%) and the fragile X mental retardation 1 (*FMR1*) gene mutation is the most common monogenic cause of ASD (2–6%) (Budimirovic and Kaufmann, 2011; Mila et al., 2018; Santoro et al., 2012). FXS is caused by transcriptional silencing of *FMR1* gene because of a trinucleotoide repeat expansion in the promoter region which results in a loss of the fragile X mental retardation protein (FMRP), an mRNA binding protein (Santoro et al., 2012). Though the mutation in the *FMR1* gene has been identified as the genetic mechanism that underlies FXS, it is not well understood how the resulting pathophysiology contributes to ID or ASD behavioral phenotypes (Mila et al., 2018; Santoro et al., 2012). Microglia have emerged as potential critical contributors to FXS pathophysiology given that postmortem analyses of FXS patients and analyses of the *Fmr1* knock-out (KO) mouse, the pre-clinical FXS mouse model, have revealed neurons with increased immature spines and spine density (Kazdoba et al., 2014; Galvez and Greenough, 2005; McKinney et al., 2005; Irwin et al., 2001). Microglia play a critical role in the development and maintenance of synapses through bidirectional communication with neurons, occurring when neurons are in distress or during neurodevelopment through the pruning process (Wu et al., 2015; Eyo and Wu, 2013; Paolicelli et al., 2011; Zhan et al., 2014). A study in *Fmr1* KO mice suggested that altered microglial engulfment of neurons might contribute to the previously established reduction in neuronal pruning (Jawaid et al., 2018). Notably, it was also demonstrated that lipopolysaccharide (LPS)-induced inflammation leads to acute elevations in pro-inflammatory cytokine expression in the hippocampus of *Fmr1* KO mouse (Hodges et al., 2020). These data imply a microglial contribution to the interaction between genetic risk factors (*FMR1* gene mutation) and environmental risk factors (infection or injury-induced inflammation). However, underlying mechanisms on how FMRP-deficient microglia respond to inflammation needs to be investigated further.

The goal of this study was to test the hypothesis that the loss of FMRP in microglia results in an altered inflammatory response, suggesting an interaction between a gene and the environment. This hypothesis was assessed by treating primary cultured microglia from brains of wild-type (WT) and *Fmr1* KO mice with LPS treatment (multiple doses and durations), then measuring pro-inflammatory cytokines (mRNA and protein), phagocytosis, and mitochondrial characteristics. Our results demonstrate that compared to WT microglia, *Fmr1* KO microglia produce elevated pro-inflammatory cytokines and elevate the phagocytic response following LPS treatment. Our experiments also revealed a basal difference in mitochondrial function and morphology between WT and *Fmr1* KO microglia, which is exaggerated with LPS stimulation. Together, these data indicate that FMRP-deficient microglia demonstrate an altered pro-inflammatory response providing evidence for an interaction between gene and environment.

## Results

### Following LPS stimulation, pro-inflammatory gene expression is significantly elevated in *Fmr1* KO microglia above expression in WT microglia

**Figure 1:**
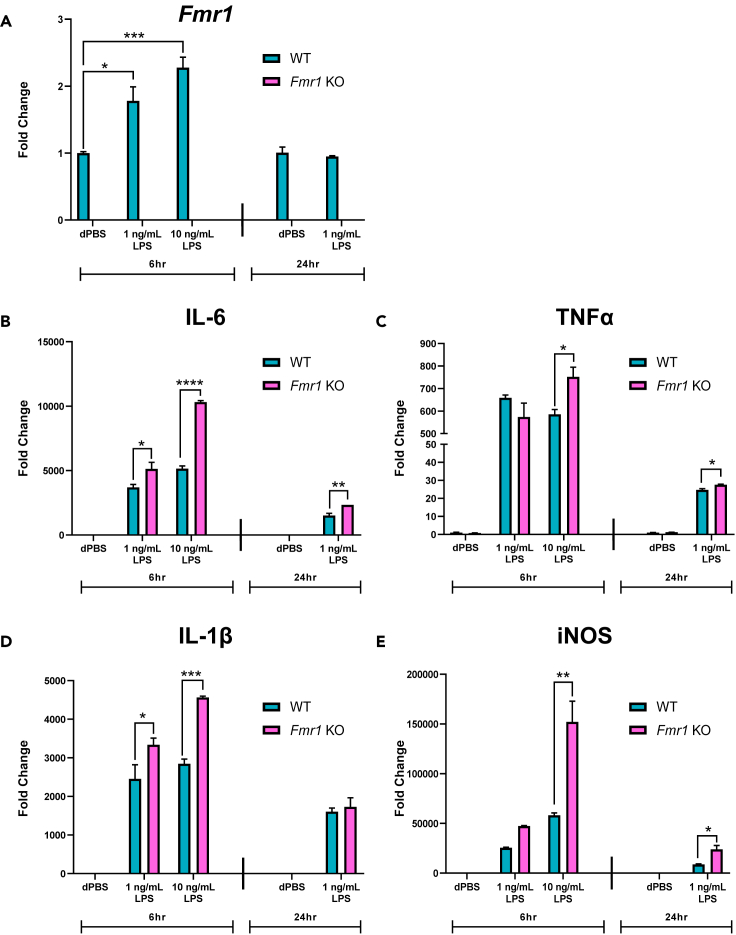
Following LPS stimulation, pro-inflammatory gene expression is significantly elevated in *Fmr1* KO microglia above expression in WT microglia (A-E) Following 6 h incubation with LPS (1 ng/mL or 10 ng/mL) and dPBS (control), or 24 h incubation with LPS (1 ng/mL) and dPBS (control), *Fmr1* expression (A), and pro-inflammatory cytokine and iNOS expression (B-E) levels were assessed in WT or *Fmr1* KO microglia using RT–qPCR. (A) Compared to dPBS treatment, 6 h 1 ng/mL LPS and 6 h 10 ng/mL LPS treatment in WT microglia increased *Fmr1* expression which was not detected in *Fmr1* KO microglia. Compared to dPBS treatment, 24 h 1 ng/mL LPS treatment in WT microglia did not change *Fmr1* expression which was also not detected in *Fmr1* KO microglia. (B) IL-6 expression was significantly elevated following 6 h 1 ng/mL LPS and 6 h 10 ng/mL LPS stimulation in *Fmr1* KO microglia compared to WT microglia. Similarly, 24 h 1 ng/mL LPS treatment elevated IL-6 expression significantly in *Fmr1* KO microglia compared to WT microglia. (C) 6 h 10 ng/mL LPS stimulation significantly up-regulated TNFα expression in *Fmr1* KO microglia compared to WT microglia. However, following 6 h 1 ng/mL LPS, there was no difference in the treatment response between *Fmr1* KO and WT microglia. 24 h 1 ng/mL LPS stimulation significantly up-regulated TNFα expression in *Fmr1* KO microglia compared to WT microglia. (D) After 6 h 1 ng/mL LPS and 6 h 10 ng/mL LPS, IL-1β expression was significantly elevated in *Fmr1* KO microglia compared to changes in WT microglia. However, 24 h 1 ng/mL LPS treatment in *Fmr1* KO microglia did not further elevate IL-1β expression. (E) 6 h 10 ng/mL LPS treatment significantly elevated iNOS expression in *Fmr1* KO microglia compared to WT microglia, whereas there was no difference in the response treatment between *Fmr1* KO and WT microglia to 6 h 1 ng/mL LPS. Following 24 h 1 ng/mL LPS stimulation, iNOS expression was significantly elevated in *Fmr1* KO microglia compared to WT microglia. n = 2–3 samples per genotype per treatment. Data are represented as mean ± SEM. Data were analyzed with a two-way ANOVA and Tukey’s multiple comparison test for post-hoc analysis. ∗p < 0.05, ∗∗p < 0.01, ∗∗∗p < 0.001, ∗∗∗∗p < 0.0001

Previous studies have suggested that microglial FMRP expression is low to almost undetectable in adult mice (Gholizadeh et al., 2015); therefore, it was necessary to determine the impact of LPS treatment on *Fmr1* gene expression in primary cultured microglia from postnatal mouse brains. Following treatment with 1 ng/mL LPS, 10 ng/mL LPS, and Dulbecco’s phosphate-buffered saline (dPBS) in cell culture medium for 6 h; or 1 ng/mL LPS and dPBS for 24 h, primary cultured microglia from brains of WT and *Fmr1* KO mice were collected and analyzed for gene expression changes using quantitative PCR with reverse transcription (RT–qPCR, see STAR Methods). Doses and treatment lengths of LPS were chosen to cover a wide range of stimulation based on previous studies demonstrating that different LPS-responsive genes are induced even at low-range doses and short durations of treatment (Huang et al., 2012). Shorter duration (2 h–6 h) typically represents the peak response of a gene to LPS treatment, while the longer duration (12 h–24 h) represents the recovery period (Huang et al., 2012). Both doses of LPS following 6 h treatment resulted in a significant increase in *Fmr1* gene expression only in WT microglia (Figure 1A, genotype x treatment, F_2,8_ = 16.61, p = 0.0014). However, there was no significant impact on *Fmr1* gene expression in WT microglia incubated with 1 ng/mL LPS for 24 h (Figure 1A). Following all treatment lengths analyzed, *Fmr1* gene expression was below the limit of detection in *Fmr1* KO microglia, independent of treatment. These data demonstrate that *Fmr1* gene expression is detectable in WT microglia, and following 6 h treatment, an inflammatory stimulus, LPS, can induce an elevation in *Fmr1* gene expression, a previously unreported observation.

Microglia, both in culture and *in vivo*, typically respond to inflammatory stimulation through the up-regulation of pro-inflammatory cytokines and other related pro-inflammatory markers (Nakamura et al., 1999; Lieb et al., 2003; Tanaka et al., 2006; Henry et al., 2008). For the purposes of this study, we determined pro-inflammatory gene expression levels for interleukin-6 (IL-6), tumor necrosis factor alpha (TNFα), interleukin-1β (IL-1β), and inducible nitric oxide synthase (iNOS). Following 6 h incubation with 1 ng/mL LPS, 10 ng/mL LPS, and dPBS; or 24 h incubation with 1 ng/mL LPS and dPBS, WT and *Fmr1* KO microglia were collected and analyzed for pro-inflammatory gene expression changes using RT–qPCR (see STAR Methods). IL-6 expression (Figure 1B, F_2,8_ = 76.45, p < 0.0001) changes in *Fmr1* KO microglia were significantly elevated when compared to changes that occurred in WT microglia following 6 h treatment with both 1 ng/mL LPS (p = 0.025) and 10 ng/mL LPS (p < 0.0001). After 1 ng/mL LPS treatment for 24 h, IL-6 expression (F_1,4_ = 23.81. p = 0.0082) was also significantly elevated in *Fmr1* KO microglia compared to expression in WT microglia (p = 0.008). TNFα gene expression (Figure 1C, F_2,8_ = 7.55, p = 0.014) response was elevated significantly in *Fmr1* KO microglia only following 10 ng/mL LPS treatment for 6 h when compared to the response in WT microglia (p = 0.036). Following 24 h treatment, TNFα gene expression in *Fmr1* KO microglia was also elevated when compared to expression in WT microglia (p = 0.019). Expression of IL-1β (Figure 1D, F_2,8_ = 16.88, p = 0.013) following 6 h treatment with both 1 ng/mL LPS and 10 ng/mL LPS was increased significantly in *Fmr1* KO microglia compared to WT microglia (p = 0.039 and p = 0.0002 respectively). However, IL-1β expression was not different between *Fmr1* KO microglia and WT microglia following 24 h treatment with 1 ng/mL LPS. Finally, iNOS gene expression (Figure 1E, F_2,8_ = 9.39, p = 0.008) following 6 h treatment was significantly higher in *Fmr1* KO microglia than WT microglia only at the 10 ng/mL LPS dose (p = 0.002). Following 24 h treatment with 1 ng/mL LPS, iNOS gene expression (F_1,4_ = 15.42, p = 0.017) was also significantly elevated in *Fmr1* KO microglia compared to WT microglia (p = 0.018). Together, these results demonstrate that compared to WT microglia, *Fmr1* KO microglia respond to LPS treatment with a greater elevation in pro-inflammatory genes (IL-6, TNFα, IL-1β, iNOS). Previous studies have demonstrated that LPS increases both pro- and anti-inflammatory cytokines (Chhor et al., 2013). Therefore, we assessed the gene expression of anti-inflammatory cytokines, transforming growth factor β (TGFβ) and interleukin 10 (IL-10) and observed no genotype effect (see Figure S1). These data suggest that the loss of FMRP expression in *Fmr1* KO microglia results in a dysregulated microglial pro-inflammatory response, the consequences of which are currently unknown.

### After LPS stimulation, pro-inflammatory cytokine secretion is significantly increased from *Fmr1* KO microglia compared to secretion from WT microglia

Microglia respond immediately to stimulation through changes in gene expression as well as the release of stored pro-inflammatory cytokines (Nakamura et al., 1999). After treatment for 6 h with 1 ng/mL LPS, 10 ng/mL LPS, and dPBS; 24 h with 1 ng/mL LPS and dPBS; 24 h with 100 ng/mL LPS and dPBS; or 48 h with 100 ng/mL LPS and dPBS, the media from both WT and *Fmr1* KO microglia were collected and analyzed for secreted pro-inflammatory cytokines accumulated in the conditioned media by enzyme-linked immunosorbent assay (ELISA, see STAR Methods). Notably, an additional higher dose (100 ng/mL) and a longer treatment length (48 h) were added to further characterize the potential cellular genotype difference in cytokine release, a process which can last longer than gene up-regulation following immune stimulation (Huang et al., 2012). Following treatment, an assessment of total cell protein was conducted using the BCA assay (see STAR Methods) to confirm that the higher dose of LPS (100 ng/mL for 24 h) did not impact the cell viability (Figure S2). Preliminary data collected (Figure S3) demonstrated that there is a range of LPS doses over which TNFα secretion increased (1 ng/mL for 6 h up to 1 ng/mL for 24 h) and a dose beyond which TNFα secretion appeared to saturate (above 1 ng/mL LPS for 24 h, Figure S3A). Though a similar pattern of increased cytokine secretion was not apparent for IL-6, with the same LPS doses and treatment durations, IL-6 did appear to reach a secretion saturation range (above 1 ng/mL LPS for 24 h, Figure S3B). A range of LPS doses below and above this apparent secretion saturation range was characterized in the pro-inflammatory cytokine secretion studies presented below.

**Figure 2:**
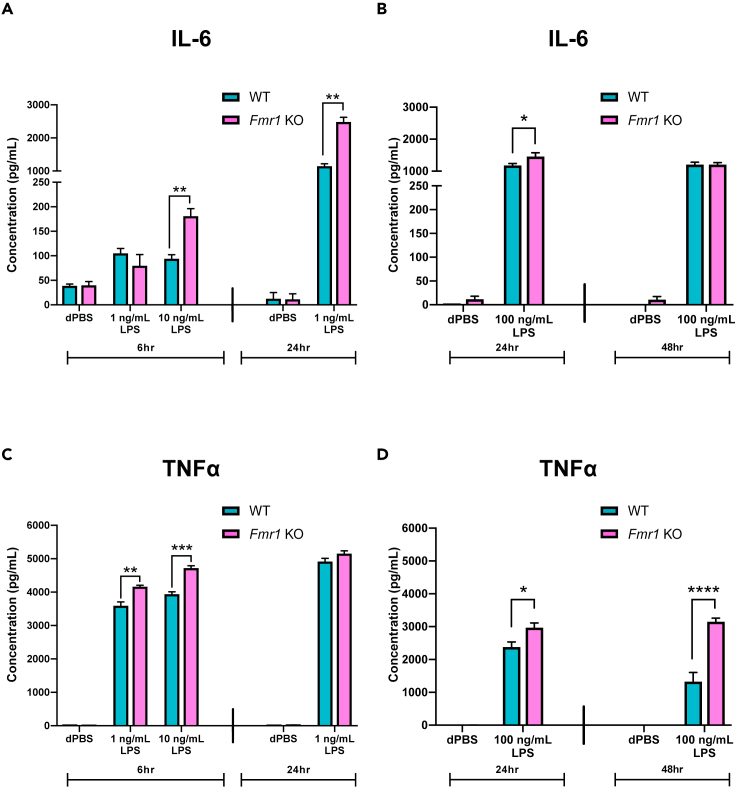
After LPS stimulation, pro-inflammatory cytokine secretion is significantly increased from *Fmr1* KO microglia compared to secretion from WT microglia Following 6 h treatment with LPS (1 ng/mL or 10 ng/mL) and dPBS (control), 24 h treatment with LPS (1 ng/mL or 100 ng/mL) and dPBS (control), or 48 h treatment with LPS (100 ng/mL) and dPBS (control), pro-inflammatory cytokine levels of *Fmr1* KO and WT microglia were assessed in cell culture media using ELISA. (A) After 6 h 10 ng/mL LPS and 24 h 1 ng/mL LPS treatment, IL-6 secretion from *Fmr1* KO micrglia was significantly elevated whereas there was no genotype difference after 6 h 1 ng/mL LPS. (B) Similarly, 24 h 100 ng/mL LPS treatment elevated *Fmr1* KO microglia IL-6 secretion above WT microglial secretion; however, there was no genotype difference following 48 h 100 ng/mL LPS stimulation. (C) TNFα protein levels were significantly increased in *Fmr1* KO microglia media treated with either 6 h 1 ng/mL LPS and 6 h 10 ng/mL LPS above WT microglia. However, there was no genotype difference in TNFα protein levels in the media after 24 h 1 ng/mL LPS incubation. (D) Following both 24 h 100 ng/mL LPS and 48 h 100 ng/mL LPS treatment, TNFα secretion was significantly elevated in *Fmr1* KO microglia media above WT microglia. n = 2–3 samples per genotype per treatment. Data are represented as mean ± SEM. Data were analyzed with a two-way ANOVA and Tukey’s multiple comparison test for post-hoc analysis. ∗p < 0.05, ∗∗p < 0.01, ∗∗∗p < 0.001, ∗∗∗∗p < 0.0001

IL-6 accumulation was significantly elevated (Figure 2A, F_2,8_ = 11.05, p = 0.005) in *Fmr1* KO media following 6 h treatment with 10 ng/mL LPS when compared to accumulation in WT media (p = 0.006). Following 24 h 1 ng/mL LPS treatment, IL-6 accumulation (Figure 2A, F_1,4_ = 70.07, p = 0.0011) was also significantly increased in *Fmr1* KO media in comparison to levels in WT media (p = 0.001). Further, following 24 h 100 ng/mL LPS treatment, IL-6 accumulation (Figure 2B, F_1,14_ = 3.56, p = 0.080) was significantly different between *Fmr1* KO media and WT media (p = 0.488). However, following 48 h LPS treatment, IL-6 accumulation (Figure 2B) was not significantly different between *Fmr1* KO and WT media. TNFα accumulation (Figure 2C, F_2,8_ = 17.02, p = 0.0013) following 6 h both 1 ng/mL and 10 ng/mL LPS treatments was significantly increased in *Fmr1* KO media compared to WT media (p = 0.0059 and p = 0.0002 respectively). However, after 24 h 1 ng/mL LPS treatment, there was no difference in TNFα accumulation between *Fmr1* KO and WT media (Figure 2C). Following 24 h 100 ng/mL LPS treatment, TNFα accumulation (Figure 2D, F_1,14_ = 5.56, p = 0.0335) was significantly elevated in *Fmr1* KO media compared to WT media (p = 0.0163). After 48 h 100 ng/mL LPS treatment, TNFα accumulation (Figure 2D, F_1,10_ = 53.45, p < 0.0001) was significantly increased in the media of *Fmr1* KO microglia when compared to WT microglia (p < 0.0001). These data support the above presented gene expression data (Figure 1) and further suggest that *Fmr1* KO microglial LPS treatment induces an elevated pro-inflammatory response.

### Following LPS treatment, *Fmr1* KO microglia phagocytose more extracellular material than phagocytosed by WT microglia

**Figure 3:**
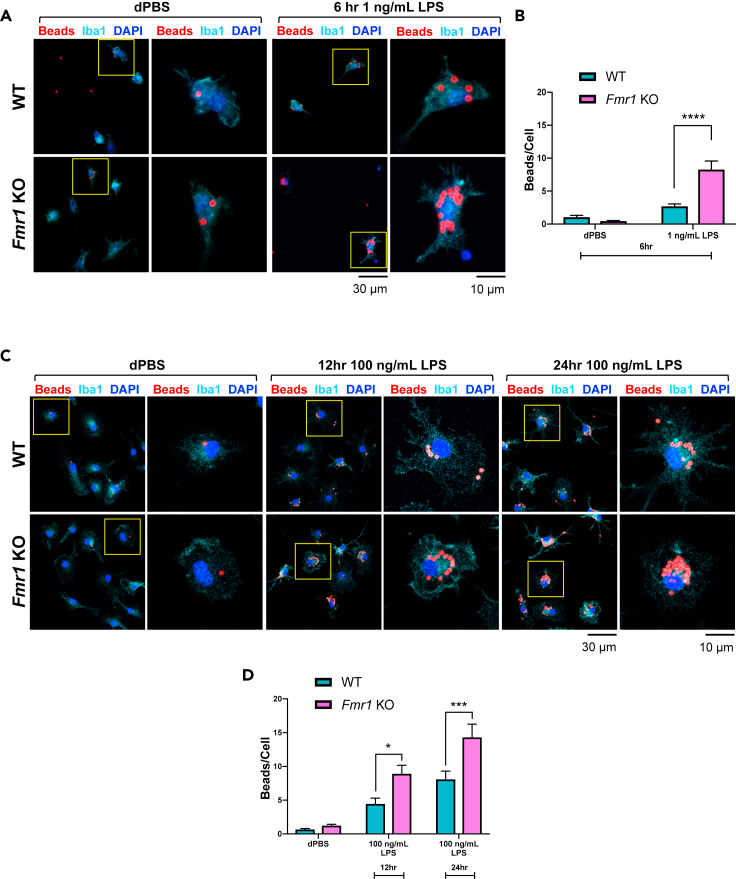
Following LPS treatment, *Fmr1* KO microglia phagocytose more extracellular material than phagocytosed by WT microglia (A and C) Following 6 h treatment with LPS (1 ng/mL) and dPBS (control), or 12 h or 24 h treatment with LPS (100 ng/mL) and dPBS (control), WT or *Fmr1* KO microglia were incubated for 2 h with fluorescent beads then phagocytosis was assessed using fluorescent imaging (A, 6 h treatment; C, 12 h/24 h treatment). Representative images from each treatment group are labeled with beads (red), microglia (Iba1, light blue), and nuclei (DAPI, dark blue). Higher magnifications of the left images (yellow boxes) are shown on the right panels. Scale bars for left panels and right panels represent 30 μm and 10 μm, respectively. (B) Quantitative data summary demonstrating that following 6 h 1 ng/mL LPS treatment, *Fmr1* KO microglia phagocytose more fluorescent beads than WT microglia. (D) Quantitative data summary demonstrating that after 12 h 100 ng/mL LPS and 24 h 100 ng/mL LPS, *Fmr1* KO microglia phagocytose more fluorescent beads than WT microglia. n = 6–8 samples per genotype per treatment. Data are represented as mean ± SEM. Data were analyzed with a two-way ANOVA and Tukey’s multiple comparison test for post-hoc analysis. ∗p < 0.05, ∗∗∗p < 0.001, ∗∗∗∗p < 0.0001

While also generating and releasing pro-inflammatory cytokines, microglia function to phagocytose dying neurons and extracellular debris during a neuroinflammatory response (Neher et al., 2011). To determine if the phagocytic response of *Fmr1* KO microglia is impacted by the loss of FMRP, WT or *Fmr1* KO microglia were incubated with 1 ng/mL LPS and dPBS for 6 h; or 100 ng/mL LPS and dPBS for 12 h or 24 h then incubated with fluorescent beads for 2 h and fixed (see STAR Methods). Images were captured to co-localize confirmed microglia (DAPI^+^;Iba1^+^) with fluorescent beads following immunostaining (Figures 3A and 3C). Following 6 h 1 ng/mL LPS treatment, *Fmr1* KO microglia engulfed more beads (Figure 3B, F_1,266_ = 36.81, p < 0.0001) when compared to WT microglia (p < 0.0001). Following treatment with 100 ng/mL LPS, *Fmr1* KO microglia also engulfed more beads than WT microglia (Figure 3D, F_2,328_ = 5.02, p = 0.0071) for both 12 h (p = 0.0177) and 24 h (p = 0.0006) durations. These data demonstrate that the loss of FMRP results in a dysregulation of microglial phagocytic activity in response to a pro-inflammatory stimulus.

### *Fmr1* KO microglia have altered mitochondrial properties and an exaggerated mitochondrial response to LPS stimulation

**Figure 4:**
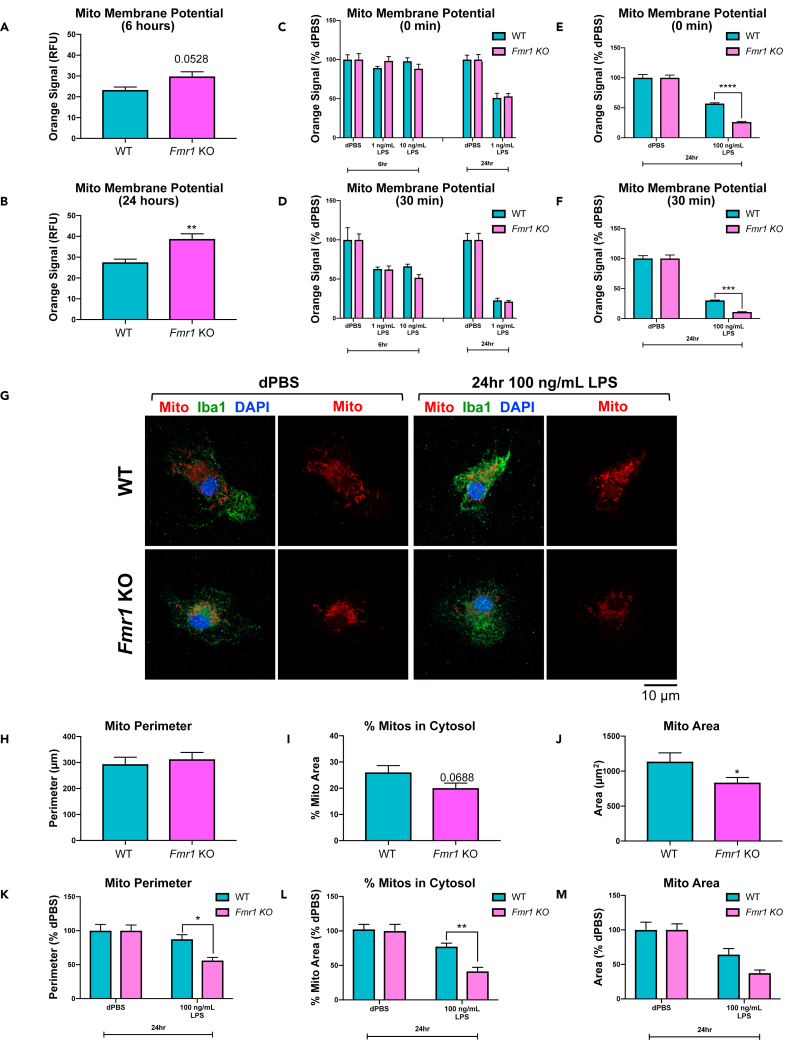
*Fmr1* KO microglia have altered mitochondrial properties and an exaggerated mitochondrial response to LPS stimulation (A-F) Following 6 h treatment with LPS (1 ng/mL or 10 ng/mL) and dPBS (control), or 24 h treatment with LPS (1 ng/mL or 100 ng/mL) and dPBS (control), *Fmr1* KO or WT microglia were assessed with MITO-ID, a live cell dye for actively respiring mitochondria that measures mitochondrial membrane potential. (A and B) The data in panels (A and B) represent only the dPBS response (basal status) in both WT and *Fmr1* KO microglia, therefore they are presented as raw data. At 6 h (A) and 24 h (B) after dPBS treatment, there was a trend toward an increase in *Fmr1* KO microglial mitochondrial membrane potential and a significantly increased *Fmr1* KO microglial mitochondrial membrane potential over WT microglia, respectively. (C–F) The data in panels (C–F) are normalized to the dPBS response to demonstrate the LPS-specific response difference. Following 6 h 1 ng/mL LPS, 6 h 10 ng/mL LPS, or 24 h 1 ng/mL LPS treatment, there was no significant difference between genotypes in the LPS-induced mitochondrial membrane potential decrease, both after 0 min MITO-ID dye incubation (C) and after 30 min dye incubation (D). However, following 24 h 100 ng/mL LPS treatment, there was an exaggerated decrease in mitochondrial membrane potential in *Fmr1* KO microglia following 0 min MITO-ID dye incubation (E) and after 30 min dye incubation (F). (G-M) Following 24 h treatment with either 100 ng/mL LPS or dPBS (control), *Fmr1* KO or WT microglia were stained with MitoTracker Red, a live cell mitochondrial membrane-dependent dye to visualize mitochondria. (G) Representative images from each treatment group with MitoTracker (red), microglia (Iba1, light blue), and nuclei (DAPI, dark blue) labeled. Scale bar represents 10 μm. (H–J) The data in panels (H–J) represent only the dPBS response (basal status) in both WT and *Fmr1* KO microglia, therefore they are presented as raw data. *Fmr1* KO microglia have no difference in mitochondria perimeter (H), have a trend toward a decrease in % mitochondria in cytosol (I), and have significantly decreased mitochondrial area (J) compared to WT microglial mitochondria. (K–M) The data in panels (K–M) are normalized to the dPBS response to demonstrate the LPS-response difference clearly. (K and L) After 24 h 100 ng/mL LPS, there is an exaggerated decrease in mitochondria perimeter (K) and % mitochondria in cytosol (L) in *Fmr1* KO microglia compared to WT microglia. (M) Following 24 h 100 ng/mL LPS, there is only a decrease in mitochondrial area because of treatment. n = 3–4 samples per genotype per treatment (MITO-ID); n = 11–13 samples per genotype per treatment (MitoTracker). Data are represented as mean ± SEM. Data were analyzed with either a one-way or a two-way ANOVA and Tukey’s multiple comparison test for post-hoc analysis. ∗p < 0.05, ∗∗p < 0.01, ∗∗∗p < 0.001, ∗∗∗∗p < 0.0001

Mitochondria increase activity during a cellular inflammatory response, undergoing fusion and fission, providing energy for increased cellular activities while also producing cytotoxic factors such as reactive oxygen species (Boje and Arora, 1992; Park et al., 2013; Roy et al., 2008). Therefore, normal mitochondrial functionality, such as maintaining the mitochondrial membrane potential and mitochondrial dynamics (fusion and fission), is necessary for a cell’s inflammatory response. Notably, inflammation increases mitochondrial membrane potential and a previous study demonstrated that LPS induces mitochondrial fission in microglia (Park et al., 2013). To better understand the genotype differences driven by the lack of the microglial FMRP, mitochondrial function and morphology in WT or *Fmr1* KO microglia were assessed by measuring mitochondrial membrane potential and cellular mitochondrial content (see STAR Methods). To measure the mitochondrial membrane potential, *Fmr1* KO or WT microglia were treated with LPS or dPBS for 6 h or 24 h then MITO-ID, a live cell dye for actively respiring mitochondria, was applied and fluorescence was measured. After 6 h treatment with 1 ng/mL LPS, 10 ng/mL LPS and dPBS; 24 h treatment with 1 ng/mL LPS and dPBS; or 24 h treatment with 100 ng/mL LPS and dPBS, mitochondrial membrane potential was measured in *Fmr1* KO or WT microglia. Measurements were collected from samples immediately following MITO-ID dye application (baseline or 0 min, Figures 4C and 4E) or 30 min (Figures 4A, 4B, 4D, and 4F) after dye application. As a result, 6 h post dPBS treatment (30 min after MITO-ID dye application), the mitochondrial membrane potential had an increased trend (Figure 4A, t_6_ = 2.41, p = 0.0528) in *Fmr1* KO microglia above WT microglia. Following 24 h dPBS incubation (30 min after MITO-ID dye application), the mitochondrial membrane potential was significantly increased (Figure 4B, t_6_ = 3.81, p = 0.0089) in *Fmr1* KO microglia above WT microglia. To better understand the impact of LPS, the mitochondrial membrane potential data collected were then normalized to dPBS within each genotype (Figures 4C–4F). After 6 h 1 ng/mL LPS, 6 h 10 ng/mL LPS, or 24 h 1 ng/mL LPS treatment, immediately following MITO-ID dye application (Figure 4C, treatment, F_4,30_ = 26.44, p < 0.0001) and 30 min following MITO-ID dye application (Figure 4D, treatment, F_4,30_ = 43.88, p < 0.0001), there was no significant difference between genotypes in the LPS-induced mitochondrial membrane potential decrease (Figures 4C and 4D). Notably, after 24 h treatment with 100 ng/mL LPS, immediately following MITO-ID dye application (Figure 4E, F_1,36_ = 19.36, p < 0.0001) and 30 min following MITO-ID dye application (Figure 4F, F_1,36_ = 8.18, p = 0.007), there was an exaggerated decrease in the mitochondrial membrane potential in *Fmr1* KO microglia compared to WT microglia (p < 0.0001 and p = 0.0010 respectively).

To visualize microglial mitochondria, *Fmr1* KO and WT microglia were treated for 24 h with 100 ng/mL LPS or dPBS then stained with MitoTracker Red, a live cell mitochondrial membrane-dependent dye. Cells were then fixed and images were captured to co-localize confirmed microglia (DAPI^+^; Iba1^+^) with MitoTracker immunostaining (Figure 4G). Using Image J and the Mitophagy macro plug-in, MitoTracker Red staining was analyzed and the following parameters were determined; (1) mitochondrial perimeter (μm), (2) % mitochondrial area within the cytosol (% mitochondria in cytosol), and (3) mitochondrial area (μm^2^). Following dPBS treatment, there was no genotype difference in microglial mitochondrial perimeter in *Fmr1* KO microglia compared to WT microglia (Figure 4H). However, following dPBS treatment, there was a decreased trend in % mitochondria in cytosol (Figure 4I, t_22_ = 1.91, p = 0.0688) and a significant decrease in mitochondrial area (Figure 4J, t_22_ = 2.12, p = 0.0452) in *Fmr1* KO microglia compared to WT microglia. To better understand the LPS treatment effect, the MitoTracker Red data collected were then normalized to dPBS within each genotype (Figures 4K–4M). Following 24 h 100 ng/mL LPS treatment, there was a significant decrease in mitochondria perimeter (Figure 4K, F_1,42_ = 4.29, p = 0.0445) in *Fmr1* KO than WT microglia (p = 0.0316). There was also an exaggerated decrease in % mitochondria in cytosol (Figure 4L, F_1,41_ = 4.96, p = 0.0314) following 24 h 100 ng/mL LPS treatment in *Fmr1* KO compared to WT microglia (p = 0.0093). However, there was no genotype difference in the decrease in mitochondrial area (Figure 4M) following 24 h 100 ng/mL LPS treatment. Together, these data demonstrate that *Fmr1* KO microglia have an elevated resting (dPBS-treated) mitochondrial membrane potential and a reduced population of mitochondria. In response to LPS treatment, *Fmr1* KO microglia have an exaggerated reduction of mitochondrial membrane potential and mitochondrial content within the cell, demonstrating a vulnerability of *Fmr1* KO microglial mitochondria to inflammation.

## Discussion

The experiments presented here were designed to determine the impact of a genetic predisposition (*Fmr1* KO) on an environmental factor (LPS-induced inflammation) demonstrated by alterations at a cellular level (microglia). The results demonstrate that *Fmr1* KO microglia *in vitro* have an elevated response to inflammation and that there are *Fmr1* KO genotype differences in microglial mitochondrial characteristics which can be exaggerated by inflammation. With LPS as the inflammatory stimulus and compared to WT microglia, activated *Fmr1* KO microglia demonstrate elevated responses in pro-inflammatory gene expression (Figure 1), cytokine release (Figure 2), phagocytosis (Figure 3), mitochondrial membrane potential (Figure 4), and mitochondrial morphology (Figure 4). These responses have previously been characterized in WT microglia *in vitro* following LPS treatment and each assessment in this study is similarly recapitulated following LPS treatment of WT microglia. Notably, our findings in this study strongly implicate that the loss of microglial FMRP results in an elevated inflammatory response, suggesting that a genetic predisposition (*Fmr1* KO) can worsen the impact of an environmental factor, inflammation, at the cellular level.

To fully characterize the LPS inflammatory response, multiple doses and incubation durations were assessed followed by the analysis of both pro-inflammatory gene expression and cytokine secretion. The previous *in vitro* study by Huang et al. (2012) demonstrated that the cellular response to LPS is both time-dependent and dose-dependent; Part of the initial inflammatory response is the up-regulation of pro-inflammatory gene transcription. While these return to baseline relatively quickly, cytokine secretion occurs secondary and is a more prolonged response to stimulation. Further, a study conducted by Kim and Li (2013) demonstrated that lower ranges of LPS doses can reliably activate microglia, whereas much higher doses can reduce viability and even induce microglial apoptosis. In our study, an extensive analysis was conducted for pro-inflammatory gene expression (Figure 1) and cytokine secretion (Figure 2) following LPS treatment with multiple doses (1 ng/mL to 100 ng/mL) and incubation durations (6 h–48 h). While the pro-inflammatory gene expression fold change in *Fmr1* KO microglia was elevated in response to all LPS treatment lengths and LPS doses analyzed, the greatest increase was observed at the doses analyzed following 6 h treatment. Specifically, only the highest dose (10 ng/mL LPS) demonstrated an increased fold change for all pro-inflammatory genes assessed in *Fmr1* KO microglia compared to WT microglia. As with pro-inflammatory gene expression responses, pro-inflammatory cytokine secretion from *Fmr1* KO microglia was consistently elevated following 6 h 10 ng/mL LPS stimulation and following the 24 h 100 ng/mL LPS stimulation. Our data presented here recapitulate the pattern recently reported in the *in vivo* study by Hodges et al. (2020). The mRNA data in this previous study demonstrated an elevated pro-inflammatory cytokine (IL-1β and IL-6) response in *Fmr1* KO mouse hippocampus at 4 h post-LPS treatment but not at 24 h post-LPS treatment (Hodges et al., 2020). Notably, analysis revealed that *Fmr1* gene expression was elevated in WT microglia following 6 h treatment with both LPS doses which was associated with the reported elevation of pro-inflammatory responses in *Fmr1* KO microglia (Figure 1). This correlation suggests that FMRP could play a role in regulating the extent of microglial response to LPS and that FMRP loss or the inability to up-regulate FMRP could contribute to the observed elevated LPS-induced *Fmr1* KO microglial responses. However, there was no induction of *Fmr1* gene expression following 24 h LPS treatment suggesting that the elevated pro-inflammatory responses observed in *Fmr1* KO microglia following 24 h LPS treatment could be due to an unresolved initial mRNA increase. These data suggest that one potential interaction is the intersection of FMRP, a regulator of mRNA translation and stability, and microglial inflammatory signaling pathways, which requires further investigation as potential future studies.

To determine if LPS treatment had a functional impact on microglia, phagocytic activity was further assessed. Multiple treatment durations (6 h, 12 h, or 24 h) and doses (1 ng/mL or 100 ng/mL) of LPS stimulation induced phagocytosis which was increased in *Fmr1* KO microglia (Figure 3). Our results demonstrate that the elevation in *Fmr1* KO microglial phagocytosis strongly correlates with the observed elevated pro-inflammatory markers in *Fmr1* KO microglia. The elevated phagocytosis response in *Fmr1* KO microglia was only observed following LPS stimulation and not prior to treatment (basal status). Therefore, it is possible that the disrupted mechanism driving this genotype difference is due to a separate mechanism than the hypothesized deficiency in pruning thought to contribute to *Fmr1* KO mice and FXS patients spine alterations (Kazdoba et al., 2014; Galvez and Greenough, 2005; McKinney et al., 2005; Irwin et al., 2001). It is also feasible that the elevated LPS response observed in *Fmr1* KO microglia implies a dysfunctional phagocytic mechanism which could then contribute to disrupted pruning (Galloway et al., 2019). However, the proposed deficit in microglial pruning is most likely dependent on neuronal interactions and signaling, which cannot be assessed when observing microglia *in vitro*. The dynamic interactions of microglia with synapses through microglia-neuron communication can be regulated by immune system molecules both under normal physiological conditions (Lee and Chung, 2019; Szepesi et al., 2018; Wohleb, 2016; Wu et al., 2015) and during inflammation (Costello et al., 2011; Wu et al., 2015; Santello and Volterra, 2012). Therefore, it is possible that an intrinsic mechanism is modulating *Fmr1* KO microglial responses to LPS stimulation, and an extrinsic mechanism is involved in microglial pruning of neuronal synapses.

Our results also showed that untreated (basal) FMRP-deficient microglia have an elevated mitochondrial membrane potential, however, LPS treatment (100 ng/mL) for 24 h resulted in a more dramatic decrease in this mitochondrial membrane potential than the decrease measured in WT microglia (Figure 4). Previous characterizations of the mitochondrial membrane potential suggest that cells with increased mitochondrial membrane potential have higher rates of transcription or translation (das Neves et al., 2010). Based on these observations, it is possible that loss of FMRP, an mRNA-binding protein, causes an increase in mitochondrial proteins in *Fmr1* KO microglia. Notably, images of *Fmr1* KO microglial mitochondria suggested that the mitochondria were smaller in area and occupied less cellular area. Following 24 h treatment with 100 ng/mL LPS, *Fmr1* KO microglial mitochondrial perimeter decreased more dramatically, indicative of shrinking mitochondria or fission which was previously observed following LPS treatment by Park et al. (2013). This previous study also demonstrated that inhibition of LPS-associated changes in mitochondrial morphology suppressed pro-inflammatory cytokine production (Park et al., 2013). Since mitochondrial functionality can further impact the cell’s energy production, calcium homeostasis, reactive oxygen species formation, and apoptosis, identifying the mitochondrial characteristics presented here represents the first step in understanding *Fmr1* KO microglial mitochondria-associated deficits.

Microglia typically function in a surveillance capacity (Nimmerjahn et al., 2005) and respond to stimuli by producing inflammatory mediators or mobilizing if necessary. Although previous studies have described abnormal microglial activation in ASD patients (Koyama and Ikegaya, 2015), this cellular phenotype has not been described in FXS patients. Abnormal microglia functioning could result in disruption of microglial pruning of neurons and of the microglial response to an inflammatory insult. Emerging literature shows that microglia are key regulators of synaptic remodeling via non-cell autonomous mechanisms in immature and adult brains (Wu et al., 2015; Fuhrmann et al., 2010; Paolicelli et al., 2011), modulated by immune signaling during basal conditions (Lee and Chung, 2019; Szepesi et al., 2018; Wohleb, 2016; Wu et al., 2015) and inflammatory conditions (Costello et al., 2011; Wu et al., 2015; Santello and Volterra, 2012). A previous study by Hodges et al. (2017) demonstrated decreased baseline expression of *Fmr1* KO hippocampal pro-inflammatory cytokines IL-6 and TNFα. Although decreased expression of pro-inflammatory cytokines suggests the potential for reduced microglial activity, these data strongly argue for an alteration in microglial homeostasis, which could detrimentally impact microglia-neuron interactions. In our study, the baseline *in vitro Fmr1* KO microglial pro-inflammatory mRNA levels were not different from WT microglial levels. This dissimilarity between studies is most likely due to the difference in cell specificity (microglia versus brain lysate) and the difference in brain region (cortex versus hippocampus). It is understood that other brain cells (neurons, astrocytes, oligodendrocytes, or endothelial cells) also contribute to the brain’s pro-inflammatory response (Skaper et al., 2018) and that microglial activation is brain-region specific (Grabert et al., 2016). In the recent study by Lee et al. (2019), a decrease in *Fmr1* KO cortical microglial population was described which could potentially explain the reduction in basal pro-inflammatory expression. As *in vitro* microglia were used in our study, cell populations were made to be equal during all experiments conducted. The data presented here provide a logical extension of previously reported studies investigating inflammation in *Fmr1* KO mice while also adding significant findings to the current understanding in this field.

Our results further provide insight into the consequences of microglial FMRP loss, specifically on an induced (LPS) inflammatory response and on microglial characteristics and function. As FMRP loss clearly drives the alterations presented here, future studies should investigate the role of FMRP binding to relevant mRNAs to unveil potential molecular mechanisms.Although no studies have specifically investigated inflammatory pathway alterations in *Fmr1* KO microglia, targets such as Toll-like receptor 4 (TLR4), the LPS receptor, and inflammatory signaling molecules nuclear factor kappa-light-chain-enhancer of activated B cells (NF-κB) and mitogen-activated protein kinase (MAPK) (Santa-Cecília et al., 2016) provide an investigative starting point. To better understand the LPS-induced phagocytic elevation, exploration of purinergic receptor (P2X7 and P2Y12) pathways (Choi et al., 2007; Webster et al., 2013), which mediate phagocytosis, should be conducted. Even though no currently published study suggests that FMRP binding can regulate purinergic pathways, Naviaux et al. (2015) demonstrated that purinergic antagonist treatment can alleviate some *Fmr1* KO mouse ASD-like features. In a study by Park et al. (2013), it was demonstrated that mitochondrial dynamin-related protein 1 (Drp1), a key regulator of mitochondrial fission could mediate the relationship between mitochondrial morphology and pro-inflammatory cytokine production. As FMRP has been shown to potentially bind mitochondrial specific mRNAs (Darnell et al., 2011), mitochondrial or bioenergetic disruptions are also of interest in expanding FXS pathophysiology understanding. Lastly, as microglia are vital to neuronal spine dynamics, forming and pruning (Miyamoto et al., 2016) and there is a well-established link between altered spine morphology and FMRP loss, future studies would need to explore the impact of microglial FMRP loss on spine morphology. Determining the impact of the *Fmr1* KO microglial alterations presented in this study is necessary to better understand the contribution of these observations to FXS pathophysiology.

### Limitations of the study

Our findings demonstrate that microglia cultured from *Fmr1* KO mice have an altered proinflammatory response, phagocytic activities and mitochondrial activities following LPS stimulation. However, the underlying molecular mechanisms of altered LPS-induced responses in FMRP-deficient microglia are not explored. Further molecular mechanism studies are needed for a better understanding of microglial contribution to FXS. In addition, all data collected in this study were from experiments with cultured microglia; therefore, further future characterizations of *in vivo* FMRP-deficient microglial responses to inflammation would contribute significant information to the understanding of FXS pathophysiology.

## STAR★Methods

### Key resources table

**Table:** 

REAGENT or RESOURCE	SOURCE	IDENTIFIER
**Antibodies**		

Anti-Iba1, Rabbit (for Immunocytochemistry)	FUJIFILM Waco Chemicals	019-19741; RRID: AB_839504
Anti-Iba1 Antibody (1022-5)	Santa Cruz Biotechnology	sc-32725; RRID: AB_667733
Cy™5 AffiniPure Donkey Anti-Rabbit IgG (H+L)	Jackson ImmunoResearch Laboratories, Inc	711-175-152; RRID: AB_2340607
Alexa Fluor® 647 AffiniPure Donkey Anti-Mouse IgG (H+L)	Jackson ImmunoResearch Laboratories, Inc	715-605-150; RRID: AB_2340862

**Chemicals, peptides, and recombinant proteins**		

DMEM (Dulbecco’s Modified Eagle’s Medium)	Corning, Inc.	10-013-CV
Fetal Bovine Serum (FBS), Regular, USDA Approved Origin	Corning, Inc.	35-010-CV
Penicillin-Streptomycin Solution, 50x	Corning, Inc.	30-001-CI
Invitrogen™ ProLong™ Gold Antifade Mountant with DAPI	ThermoFisher Scientific	P36931
Gibco™ Distilled Water	ThermoFisher Scientific	15-230-147
Gibco™ DPBS (dPBS), w/calcium, w/magnesium,	ThermoFisher Scientific	14-040-133
Invitrogen™ TRIzol™ Reagent	ThermoFisher Scientific	15596018
Deoxyribonuclease I (DNase) from bovine pancreas	MilliporeSigma	11284932001
Lipopolysaccharides (LPS) from *Escherichia coli* O127:B8, purified by phenol extraction (Lot # 037M4067V)	MilliporeSigma	L3129
Latex beads, carboxylate-modified polystyrene, fluorescent red, aqueous suspension, 0.5 μm mean particle size	MilliporeSigma	L3280-1ML
Applied Biosystems™ PowerUp™ SYBR™ Green Master Mix	ThermoFisher Scientific	A25741
Invitrogen™ MitoTracker™ Red CMXRos - Special Packaging	ThermoFisher Scientific	M7512
Parafromaldehyde (PFA), 96%, extra pure, ACROS Organics™	ThermoFisher Scientific	41678-0010
Falcon® 70 μm Cell Strainer, White, Sterile, Individually Packaged, 50/Case	Corning, Inc.	352350
Fluriso™, Isoflurane, USP	VetOne	502017
10X PBS	ThermoFisher Scientific	BP3994
D(+)-Sucrose, 99.7%, for biochemistry, ACROS Organics™	ThermoFisher Scientific	AC177140010

**Critical commercial assays**		

Pierce™ BCA Protein Assay Kit	ThermoFisher Scientific	23225
Mouse IL-6 DuoSet ELISA	R&D Systems, Inc.	DY406
Mouse TNF-alpha DuoSet ELISA	R&D Systems, Inc.	DY410
MITO-ID® Membrane potential detection kit	Enzo Life Sciences	ENZ-51018-0025
Applied Biosystems High-Capacity cDNA Reverse Transcription Kit	ThermoFisher Scientific	4368814

**Experimental models: Organisms/strains**		

Mouse: FVB.129P2-*Pde6b*^*+*^*Tyr*^*c-ch*^/AntJ	Jackson Laboratory	004828; RRID: IMSR_JAX:004828
Mouse: FVB.129P2-*Pde6b*^+^*Tyr*^*c-ch*^*Fmr1*^*tm1Cgr*^/J	Jackson Laboratory	004624; RRID: IMSR_JAX:004624

**Oligonucleotides**		

Custom DNA Oligos, Standard DNA Oligos, see Table S2	MilliporeSigma	OLIGO

**Software and algorithms**		

GraphPad Prism	GraphPad	N/A

### Resource availability

#### Lead contact

Further information and requests for resources and reagents should be directed to and will be fulfilled by the lead contact, Hye Young Lee (leeh6@uthscsa.edu).

#### Materials availability

The study did not generate new unique reagents.

### Experimental model and subject details

#### Animals

WT mice (stock #004828, FVB.129P2-*Pde6b*^*+*^ *Tyr*^*c-ch*^/AntJ), and *Fmr1* KO mice (stock #004624, FVB.129P2-*Pde6b*^+^ *Tyr*^*c-ch*^ *Fmr1*^*tm1Cgr*^/J) were obtained from Jackson Laboratory. These *Fmr1* KO mice were originally designed using a neomycin resistance cassette targeted to exon 5 of the *Fmr1* gene as previously reported ('*Fmr1* knockout mice: a model to study fragile X mental retardation. The Dutch-Belgian Fragile X Consortium' Author Anonymous, 1994). The use and care of animals in this study follow the guidelines of the University of Texas Health Science Center at San Antonio (UTHSCSA) Institutional Animal Care and Use Committee. Breeding cages (male mouse and two female mice) were maintained to generate pregnant females for primary glial cultures. Females were checked daily for the presence of a plug to determine the exact date of pregnancy and to calculate when litters would be born. Any health concerns or issues were handled by the veterinary staff maintained with UTHSCSA in the Department of Lab Animal Resources.

#### Primary glial cultures

To generate mixed glial cultures, male and female pups were sacrificed on postnatal day 2 (PND2) at a 1:1 male: female sex ratio then the cortex was isolated. The brain tissue of 4 pups of the same genotype, WT and *Fmr1* KO, were pooled to create a cortical mixed glial culture using previously established methods (Floden and Combs, 2007). Briefly, following dissection, cortical tissue was trypsinized and treated with DNase, homogenized, filtered through a cell strainer, pelleted in centrifuged, then resuspended in warm media (DMEM, 10% FBS, 1X penicillin-streptomycin) before plating. 24 h following the initial plating or 1 day *in vitro* (DIV), the media were changed to remove any remaining debris and to encourage cellular growth.

### Method details

#### Microglia collection and experimental conditions

After 8–10 DIV, glial cultures were ‘shaken’ at 230 rpm for 3.5 h then microglia cells suspended in the media were collected, counted and plated for subsequent experiments. Previous work by Floden and Combs (2007) demonstrated that while it is possible to successfully harvest microglia following repetitive ‘shake-offs’ from a single glial culture, the magnitude of responsiveness to LPS treatment decreases with each shake-off. To minimize this impact on our experiments, we only compared data collected from the same experiment and ‘shake-off’, then we compared fold changes of the LPS-induced cytokine production to confirm similar magnitude of responses in WT microglia. 24 h post plating, microglial health was confirmed prior to replacing media with treatment media containing either saline or LPS at increasing doses (1 ng/mL, 10 ng/mL, 100 ng/mL) for varying lengths of time (6, 12, 24, 48 h) depending on the experiment. This was accomplished for both genotypes, WT and *Fmr1* KO. A summary of the experimental conditions, including treatment doses, durations, and assessments, are described in Table S1.

#### Quantitative PCR with reverse transcription (RT–qPCR)

For experiments assessing changes in gene expression following LPS stimulation, WT and *Fmr1* KO microglia were cultured in a 12-well plate a density of 1.6 × 10^5^ cells/well. At the end of the treatment duration (see Table S1), the media were collected (for analysis see: Cytokine assessment below) and TRIzol was added directly to the cultured microglia then mRNA was isolated according to manufacturer instructions. mRNA integrity and concentration were determined using a nanospectrophotometer (NanoDrop One, ThermoFisher Scientific, Waltham, MA) then a cDNA library was created using Applied Biosystems High-Capacity cDNA Reverse Transcription Kit (ThermoFisher Scientific). cDNA and gene-specific primers (*Fmr1, Il6, Tnfα, Il1β, Nos2, Tgfb1, Il10*) were used in real-time PowerUp SYBR Green qPCR reactions (Applied Biosystems) to quantify changes in gene expression following LPS treatment in microglia cells. Data collected (ct values) were used to calculate fold changes (delta-delta-ct) in gene expression relative to WT-dPBS treated microglia at the various time-points tested (Figures 1 and S1). The *Ppia1* gene was used as an internal housekeeping gene in the calculation of fold changes. Primer sequences can be found in Table S2.

#### Cytokine assessment

To quantify the release of cytokines in microglia following LPS treatment, the media from the experiments described above were collected then IL-6 and TNFα protein was measured using DuoSet ELISA kits (R&D Systems, Inc. Minneapolis, MN) according to manufacturer instructions. Media were also collected from WT and *Fmr1* microglia cultured in a 96-well plate at a density of 2.0 × 104 cells/well following additional treatment conditions (see Table S1). Cytokine concentrations in the media samples were read using a BioTek Synergy HT microplate reader (Winooski, VT) and calculated based on a standard curve (Figures 2 and S3).

#### Total protein concentration

To determine if the high LPS dose (100ng/mL) impacted the viability of the cells during treatment, the total cellular protein concentration was assessed after 24 h following collection with a lysis buffer. The BCA protein assay (ThermoFisher Scientific) was completed according to the manufacturer instructions and samples were read using a BioTek Synergy HT microplate reader (Winooski, VT) and calculated based on a standard curve (Figure S2).

#### Phagocytosis

The functional impact of LPS treatment on WT and *Fmr1* KO microglia was determined using a fluorescent bead phagocytosis assay. Microglia were cultured on glass coverslips in a 12-well plate and treated with LPS (see Table S1) then microglia where incubated with fluorescent beads (Sigma, St. Louis, MO) for 2 h at 37°C. Following incubation, microglia were washed thoroughly then were fixed with 4% PFA. Coverslips were immunostained for microglia (with anti-Iba1) and nuclei (DAPI) prior to being mounted on a glass slide. Microglia, DAPI and beads were imaged using Zeiss Apotome and Zeiss Confocal microscope. Beads per cell (microglia) were recorded and averaged per treatment group (Figure 3). For each genotype, treatment and time-point, 2–3 coverslips (1 coverslip/well) were plated and stained for analysis. Following staining, each coverslip was imaged, and 2–3 images were collected for quantification. Within each image, every microglial cell was assessed for co-localization of beads (i.e. 0+ beads). All beads per cell (microglia) values were averaged for each treatment or time-point and statistically compared between genotypes.

#### Mitochondria function (MITO-ID and MitoTracker)

MITO-ID (Enzo Biochem, New York, NY), a live cell dye for actively respiring mitochondria was used to assess mitochondrial membrane potential and MitoTracker Red (Invitrogen, Waltham, MA), a live cell mitochondrial membrane-dependent dye, was used to image mitochondria (Figure 4). Following LPS treatment (see Table S1), microglia were incubated for 30 min with MITO-ID as per kit instructions. Then microglia were imaged immediately using the microplate reader followed by an additional 30 min incubation at 37°C then imaged a second time. Following LPS treatment (see Table S1), microglia were incubated for 10 min with 100 nM MitoTracker Red, then cells were fixed. Microglia were imaged using Zeiss confocal microscope and analyzed using Mito-Morphology Macro for FIJI as previously described (Wiemerslage and Lee, 2016). Briefly, MITO-ID images were uploaded into FIJI and the Mito-Morphology Macro was used to analyze the image for mitochondrial characteristics. Both the MITO-ID and MitoTracker data are initially presented as raw data to compare the dPBS response data between WT and *Fmr1* KO microglia. To clearly demonstrate the LPS-induced response, the data are then presented as normalized to the dPBS response for each genotype.

### Quantification and statistical analysis

One-way and two-way ANOVA were used for the statistical analysis followed by a Tukey's post hoc analysis when there was a significant interaction or main effect. All the statistics showed that variances are similar between the groups that are being statistically compared. No statistical methods were used to pre-determine sample sizes; however, all sample sizes are similar to those generally used in the field. Sample size (n) is indicated in each figure legend. Statistical significance is shown as follows: ∗p < 0.05, ∗∗p < 0.01, ∗∗∗p < 0.001, ∗∗∗∗p < 0.0001. p > 0.05 was considered as not significant.

## Data Availability

• All data reported in this paper will be shared by the lead contact upon request. • This paper does not report original code. • Any additional information required to reanalyze the data reported in this paper is available from the lead contact upon request.
