# Supplementary material for: Altered inflammatory response in FMRP-deficient microglia
Source: iScience. 2021 Oct 15;24(11):103293. doi: 10.1016/j.isci.2021.103293 (PMC8602000; doi:10.1016/j.isci.2021.103293)
Supplement: Document S1. Figures S1–S3 and Tables S1 and S2 [file mmc1.pdf]

iScience, Volume *24*

Supplemental information

Altered inflammatory response in FMRP-deficient microglia

Jennifer M. Parrott, Thomas Oster, and Hye Young Lee

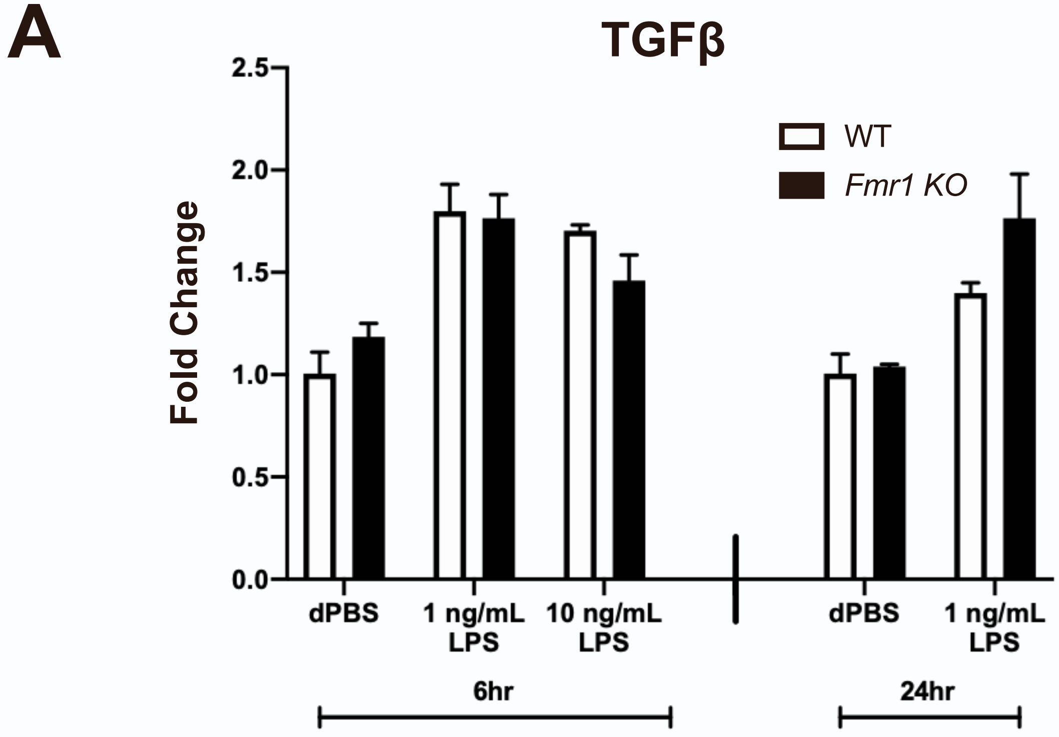

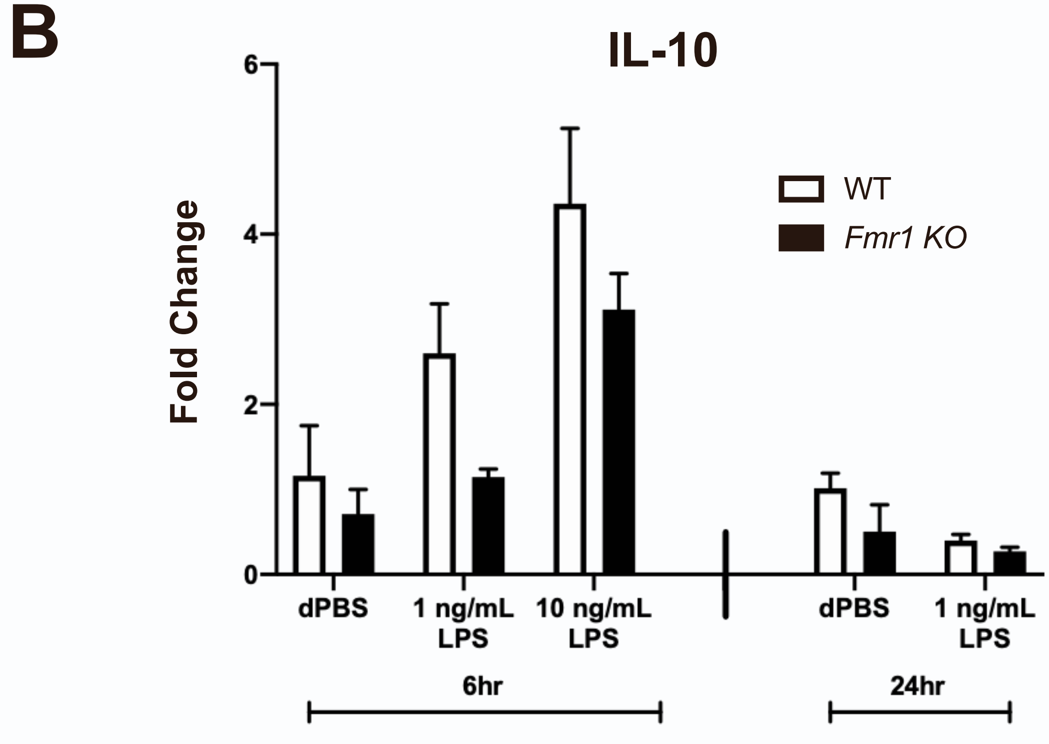

Figure S1. Microglial anti-inflammatory gene expression following LPS treatment. Related to Figure 1. (A and B) Following 6 h or 24 h treatment with 1 ng/mL LPS, 10 ng/mL LPS or dPBS (control), cells were collected from WT and *Fmr1* KO microglia and anti-inflammatory cytokine gene expressions (TGFβ and IL-10) were assessed using RT-qPCR. (A) At all doses and treatment durations, LPS significantly elevated TGFβ mRNA, however there was no difference between genotype responses. (B) IL-10 mRNA was increased by the 6 h LPS treatments and reduced by the 24 h 1 ng/mL LPS treatment. Again, there was no genotype difference in the impact of LPS on IL-10 gene expression. n = 2-3 samples per genotype per treatment. Data are represented as mean ± SEM. Data were analyzed with a one-way ANOVA and Tukey's multiple comparison test for post-hoc analysis.

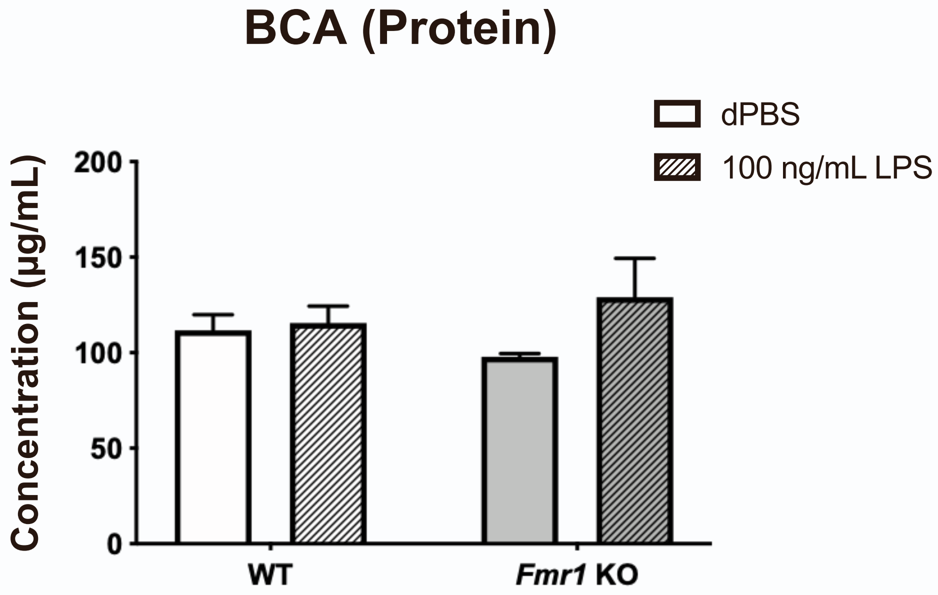

Figure S2. Total protein quantification following 24 h 100 ng/mL LPS treatment. Related to Figure 2. Following 24 h treatment with 100 ng/mL LPS or dPBS (control), cells were collected from WT and *Fmr1* KO microglia and total protein concentration (µg/mL) was determined using the BCA assay. There was no impact of genotype or LPS treatment on total protein concentration. n = 3-5 samples per genotype per treatment. Data are represented as mean ± SEM. Data were analyzed with a one-way ANOVA and Tukey's multiple comparison test for post-hoc analysis.

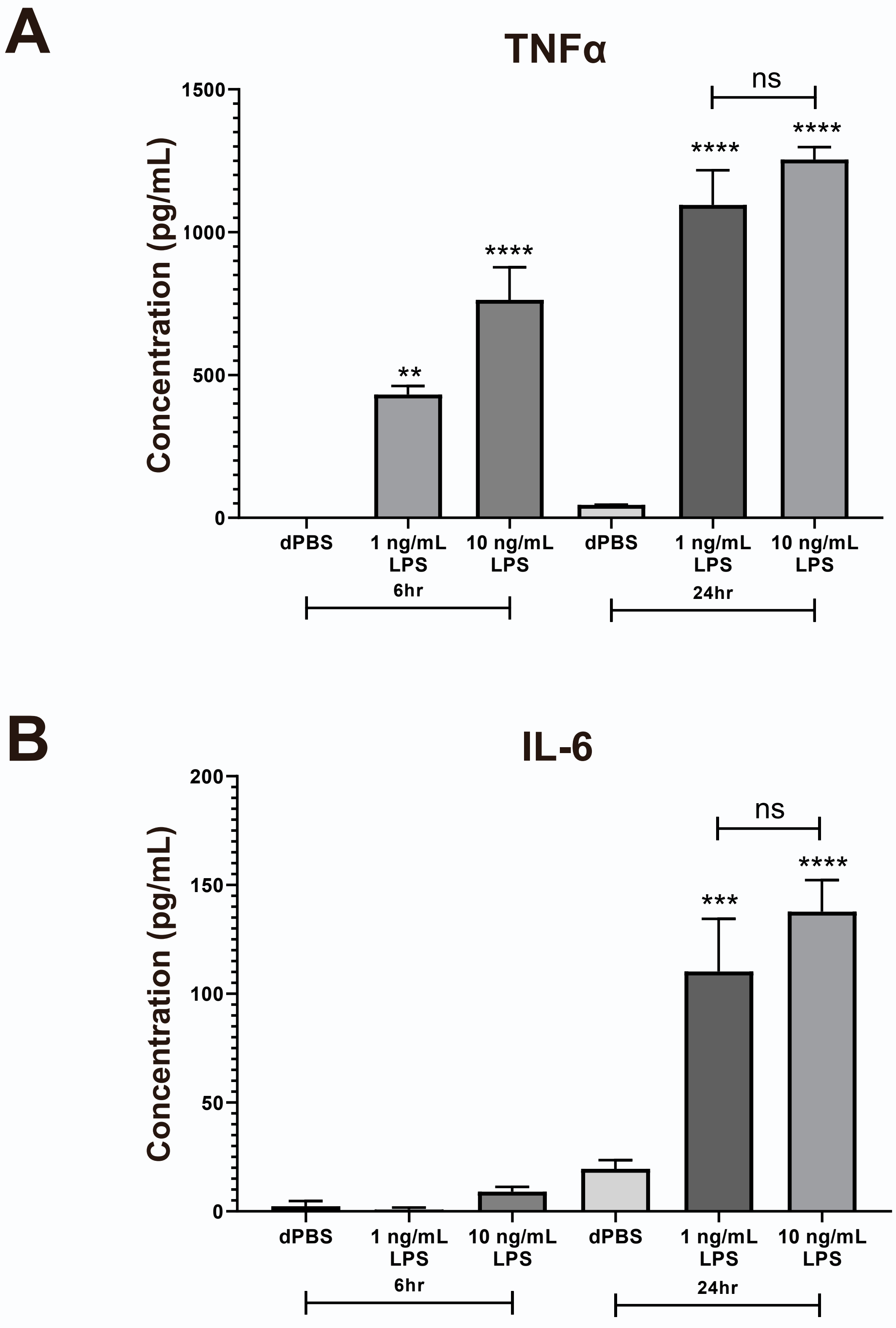

Figure S3. Impact of various LPS doses and durations on pro-inflammatory cytokine secretion. Related to Figure 2. (A-B) Following 6 h or 24 h treatment with 1 ng/mL LPS, 10 ng/mL LPS or dPBS (control), media were collected from WT microglia and pro-inflammatory cytokines (TNFα and IL-6) were assessed using ELISA. (A) At all doses and durations, LPS significantly elevated TNFα secretion. (B) IL-6 was only increased by both 24 h LPS treatments (1 ng/mL and 10 ng/mL). n = 2-3 samples per genotype per treatment. Data are represented as mean ± SEM. Data were analyzed with a one-way ANOVA and Tukey’s multiple comparison test for post-hoc analysis. **$p<0.01$, ***$p<0.001$, ****$p<0.0001$

Assessment	Dose	Duration
Gene expression (**Figure 1 and S1**)	1 ng/mL LPS	6 and 24 hr
	10 ng/mL LPS	6 hr
Cytokine secretion (**Figure 2**)	1 ng/mL LPS	6 and 24 hr
	10 ng/mL LPS	6 hr
	100 ng/mL LPS	24 and 48 hr
Phagocytosis (**Figure 3**)	1 ng/mL LPS	6 hr
	100 ng/mL LPS	12 and 24 hr
MITO-ID (**Figure 4**)	1 ng/mL LPS	6 and 24 hr
	10 ng/mL LPS	6 hr
	100 ng/mL LPS	24 hr
MitoTracker (**Figure 4**)	100 ng/mL LPS	24 hr
Total protein (**Figure S2**)	100 ng/mL LPS	24 hr
Cytokine secretion (**Figure S3**)	1 ng/mL LPS	6 and 24 hr
	10 ng/mL LPS	6 and 24 hr

Table S1. Experimental treatment scheme. Related to Figures 1, 2, 3, 4, S1, S2 and S3. A summary of the experimental conditions, including treatment doses, durations, and assessments are listed.

Gene	Sequence
Ppia1 (forward)	GAGCTGTTTGCAGACAAAGTTC
Ppia1 (reverse)	CCCTGGCACATGAATCCTGG
Fmr1 (forward)	GTGGTTAGCTAAAGTGAGGATGAT
Fmr1 (reverse)	CAGGTTTGTTGGGATTAACAGATC
Il6 (forward)	ACCACTTCACAAGTCGGAGGCT
Il6 (reverse)	TCTGCAAGTGCATCATCGTTGT
Tnfα (forward)	CATCAGTTCTATGGCCCAGA
Tnfα (reverse)	TGCTCCTCCACTTGGTGGTT
Il1β (forward)	TGTAATGAAAGACGGCACACC
Il1β (reverse)	TCTTCTTTGGGTATTGCTTGG
Nos2 (forward)	CAGCTGGGCTGTACAAACCTT
Nos2 (reverse)	CATTGGAAGTGAAGCGTTTCG
Tgfb1 (forward)	TGATACGCCTGAGTGGCTGTCT
Tgfb1 (reverse)	CACAAGAGCAGTGAGCGCTGAA
Il10 (forward)	GCCAAGCCTTATCGGAAATG
Il10 (reverse)	CACCCAGGGAATTCAAATGC

Table S2. Primer oligonucleotide sequences. Related to Figures 1 and S1. Primer sequences used for RT-qPCR are listed.
